# Organizing Telemonitoring—Decision-Making Between Centralized and Distributed Models in the Netherlands, Using the Non-Adoption, Abandonment, Scale-Up, Spread, and Sustainability (NASSS) Framework: Case Study

**DOI:** 10.2196/69349

**Published:** 2025-10-08

**Authors:** Nienke Antine Elferink, Manon Jacqueline Roest, Anne Marie Weggelaar, Marleen de Mul

**Affiliations:** 1 Erasmus School of Health Policy & Management, Erasmus University Rotterdam, Burgemeester Oudlaan 50, Rotterdam, 3062 PA, The Netherlands, 31 104081868; 2 Tranzo, Tilburg University, Tilburg, The Netherlands; 3 Clinical Informatics, Eindhoven University of Technology, Eindhoven, The Netherlands

**Keywords:** telemonitoring, centralized telemonitoring, distributed telemonitoring, NASSS framework, qualitative study

## Abstract

**Background:**

Telemonitoring can be implemented using either centralized or distributed organizational models. However, few published studies explore which conditions make one model preferable over the other, or how to choose between these two.

**Objective:**

This study aimed to investigate the decision-making factors across several domains (eg, technological, personal, and organizational) when selecting the telemonitoring model.

**Methods:**

We conducted a multiple case study across 4 purposively sampled hospitals to gain a range of perspectives on organizational models for telemonitoring. Selection criteria included: (1) type of organizational model, (2) type of collaborating partners, (3) task division of handling notifications, and (4) it had to be implemented at scale, rather than being in an exploratory phase. Data was collected in a document study, 13 semistructured interviews, and a focus group. The topic list was based on the domains of the NASSS (non-adoption, abandonment, scale-up, spread, and sustainability) framework. Interviewees (n=13) were 5 project leaders, 2 tele-nurses, 4 health care professionals, and 2 clinical informaticians. Data analysis was performed iteratively and included reflective thematic analysis. A member-checking focus group was organized to verify and reflect on the findings.

**Results:**

Various preferential factors based on the seven domains of the NASSS framework were explored for both centralized and distributed telemonitoring models: (1) Condition: the choice of objective, usually based on organizational strategy, determines whether telemonitoring will be centralized or distributed. (2) Technology: the preference for a model is determined by the anticipated number of notifications the application generates for a specific patient group. (3) Value proposition: the perceived cost-effectiveness and overall value to the patient shape the value proposition for each model. (4) Adopters: the new role of tele-nurse emerged in centralized monitoring centers (CMCs), necessitating new competencies, task redistribution, and shifts in responsibility. The importance of trust among staff became evident in the context of task redistribution. (5) Organization: CMCs are typically organized regionally, in partnerships or network arrangements, which can be time-consuming yet offer significant potential for impact. (6) Wider system: The existing Dutch reimbursement system does not incentivize CMCs because the payment structure is still based on a per-treatment model. (7) Adaptation over time: with advancements in technology, including artificial intelligence, organizing telemonitoring through CMCs is likely to gain popularity.

**Conclusions:**

Our study highlights that when decision makers are choosing which telemonitoring model—centralized or distributed—to implement in their organization, deciding on the suitability of the model depends on multiple contextual factors. Our findings illustrate that decisions made for patient group selection, technology design, and value proposition significantly influence each other. It is therefore crucial for decision makers to understand these interactions and corresponding dynamics to better align their strategies with the operational realities of their organization.

## Introduction

Telemonitoring outpatient and inpatient care (also coined remote patient monitoring, telehealth, and telemedicine) is a recognized solution to address health care challenges such as rising costs, staff shortages, and the need to improve cost-effectiveness, treatment quality, and reduce waiting times [1-4]. Telemonitoring is defined as “the use of information technology to monitor patients at a distance” [5]. Telemonitoring enables clinicians to maintain and adjust their patients’ plan of care by using remotely gathered data, such as vital signs, to proactively make medical decisions about a patient’s care [6]. It involves the collection, transmission, evaluation, and communication of individual health data from patients to health care providers using technology [7]. Because telemonitoring is widely touted to improve patient care while reducing staff workload and costs, governments worldwide stimulate it as a paramount innovation [8,9]. While much research has focused on the positive outcomes of telemonitoring for patients and organizations [10-12], less attention has been given to the organizational aspect of telemonitoring. Specifically, the considerations for various organizational models—centralized, distributed, or a combination of both [13-15]. However, which model is preferable in a given context and how to choose between the models remains unclear.

In general, 2 organizational models are in place. First, a centralized organizational model, which places patient monitoring in a physical or virtual centralized monitoring center (CMC) and is primarily conducted by tele-nurses rather than physicians. CMC can involve monitoring patients across an entire health care organization, network, or even a region [13,16,17]. The literature reports notable advantages to a centralized organizational model, including improved efficiency and cost-effectiveness through economies of scale and pooled resources, which are especially beneficial in rural areas and alleviate staff shortages [18,19]. The larger volume of cases in centralized telemonitoring permits 24/7 operation, instead of being confined to office hours in outpatient clinics [8,20]. Since 2019, structural financing for remote care (telehealth) was established in the Netherlands. Under specific conditions and for designated diagnoses, hospitals became eligible to claim reimbursement for remote care through the existing diagnosis treatment combination system [9]. This reimbursement facilitated the digital transformation of health care, aiming for more efficiency in health care delivery. Recent studies have shown that patients are satisfied with centralized telemonitoring [8,21]. Most professionals view centralized telemonitoring as favorable, although some are less acquainted with telemedicine and harbor reservations [8,22]. The second is a distributed organizational model in which professionals monitor their specific patient population [15]. Professionals believe they possess a better contextual understanding of their patients’ data than tele-nurses who do not know a specific patient [18]. Some studies have shown that the doctor-patient relationship enhances patients’ adherence to telemonitoring restrictions and therapy agreements. However, studies on the differences between centralized and distributed organizational models are lacking [13,15,18].

Numerous studies, including several literature reviews, have explored the implementation of telemonitoring for patients with diseases such as hypertension, heart failure, diabetes, and chronic obstructive pulmonary disease (COPD) [eg, 10,23,24]. These studies highlight various organizational models and the challenges associated with the complexity of implementing telemonitoring [25]. However, the challenges of organizing telemonitoring extend beyond adoption or implementation obstacles. They require a deeper exploration of different aspects, including context variables at the micro, meso, and macro levels [26,27].

This is particularly relevant, as both centralized and distributed organizational models for telemonitoring can be implemented in a single organization or across multiple organizations, potentially spanning the entire care chain [26]. To address this complexity, the NASSS (non-adoption, abandonment, scale-up, spread, and sustainability) framework provides a comprehensive lens, through which 7 key domains are examined: condition, technology, value proposition, the adopter’s perspective, organizational context, the wider context, and adaptation over time [28]. Importantly, the framework does not imply a linear relationship between the domains but instead emphasizes their interconnectedness and mutual influence [29]. With this framework, barriers and facilitators for implementation can be identified, and the complexity of each domain can be assessed [30,31]. Given this, our research question is as follows: which considerations are taken into account when choosing between centralized or distributed telemonitoring, following the NASSS framework?

## Methods

### Study Design

Using a qualitative research approach, we conducted a multiple case study to explore the factors hospitals consider when choosing between centralized and distributed telemonitoring models. The study complied with the COREQ (Consolidated Criteria for Reporting Qualitative Research; see Checklist 1) [32]. The first author is a medical student in health sciences; the other authors are experienced researchers (PhD, associate professor, and endowed professor).

### Ethical Considerations

Ethical approval was secured from the Ethics Review Board of Tilburg University (ReferenceTSB_RP741). Data storage is compliant with the General Data Protection Regulation.

### Setting

This study was executed in the Netherlands. This country has been implementing a series of policy measures aimed at facilitating the digital transformation of health care since 2012. Measures include the introduction of incentive grants, national guidelines, specific volume targets for hospitals, and structural financing for remote care (telehealth encompassing telemonitoring) [33-35]. In the face of staff shortages, this has led to new forms of (hybrid) care that aim to reduce the number of follow-up outpatient clinic visits for chronic patients, reduce hospital length of stay after surgery, etc. The Netherlands manages the health system through self-regulation among private entities: health care services are provided by private providers and managed by private health insurers. Every individual in the Netherlands has the option to select their health care insurer and health care plan [36]. Since 2019, hospitals are eligible to claim reimbursement for remote care under specific conditions and for designated diagnoses, through the existing Diagnosis treatment combination (comparable to Diagnosis Related Group) system, with costs covered under the statutory basic health insurance package. Hence, telemonitoring is covered by the Dutch health insurance system [9]. In the Netherlands, the availability of high-speed broadband internet and 4G mobile network is high, and an estimated 90% of the Dutch people use this on a daily basis. Still, health care organizations do not have the funds to upscale telemonitoring and therefore implementation progresses slowly.

Within this context, a purposive sample of teaching hospitals based on 2 organizational models (ie, centralized monitoring and distributed monitoring) was selected. (See MultimediaAppendices 1  2 for a visual representation of the centralized and distributed telemonitoring models). Hospitals using telemonitoring were selected from a network of Dutch teaching hospitals (Samenwerkende Topklinische Ziekenhuizen). Given the relatively small number of hospitals in the Netherlands (69 nonprofit hospital organizations), and the fact that only a few have implemented telemonitoring at scale, due to its relatively innovative nature, the selection of potential participants was constrained. To ensure comprehensive representation, all (12/12) early adopters of telemonitoring were put on a list. Project leaders responsible for telemonitoring in these hospitals were approached via email, which detailed the aim and design of this study. A total of four hospitals declined to participate (4/12,33%) citing as reasons: time constraints or maternity leave. Of the hospitals willing to participate (8/12, 67%), we purposely sampled that met the inclusion criteria outlined in Table 1, striving for a maximum variety of cases. An additional criterion was that the telemonitoring program had to be implemented at scale, rather than being in a pilot or exploratory phase.

We included 2 hospitals with primarily centralized telemonitoring (A and C) and 2 hospitals with predominantly distributed telemonitoring models (hospitals B and D). While hospitals B and D incorporated some centralized elements (300/3000, 10% and 1000/11,000, 9%, respectively, of their telemonitoring was conducted through a central unit) the vast majority (2700/3000, 90%) were organized in a distributed manner across care providers. In addition, in terms of collaborative partnerships, we included hospitals that operated independently without external collaboration (hospital A), those that collaborated with general practitioners in organizing telemonitoring (hospital B), and those that partnered with other hospitals (hospitals C and D). Finally, regarding role division in the monitoring process, we included hospitals where tele-nurses (hospitals A, B, and C), nurse practitioners (hospitals B and D), and residents (hospital D) were responsible for viewing telemonitoring alerts. Table 1 shows this overview. While the 2 distributed cases (hospitals B and D) also implemented centralized elements, they were classified as predominantly distributed.

**Table 1.** An overview of the hospitals included on the basis of the selection criteria.

Criterion	Hospital A	Hospital B	Hospital C	Hospital D
Organizational structure	Centralized	Predominantly distributed	Centralized	Predominantly distributed
Number of telemonitored patients	2000	3000 of which 300 (10%) centralized and 2700 distributed.	500	11,000 of which 1000 (9%) centralized and 10,000 distributed.
Partnerships	None	General practitioners	Hospital network	Neighbor hospital
Role division of handling telemonitoring alerts	Tele-nurses	Tele-nurses (both nurses and nurse practitioners)	Tele-nurses (nurses with physical disabilities)	Tele-nurses (both nurses and nurse practitioners); residents during outside-of-office hours.

### Data Collection

Data collection included a document study, 13 interviews conducted between March and May 2023, and a member-checking focus group held in May 2023. The researchers began by requesting implementation-related documents (eg, implementation plans, current-state analyses, vision-related documents, or briefing materials for executive boards) from the project leaders of all 4 hospitals to study the organizational structures and challenges. Next, a researcher (NAE) conducted 13 semistructured interviews lasting between 30 and 60 minutes (see Table 2 for participant characteristics). The respondents were recruited via the project leader based upon criteria set by the researcher, including knowledge of telemonitoring and the respondent’s involvement in the implementation process. Informed consent was obtained before the interview. The participants represented a diverse group of health care professionals and the project leaders, including a clinical informatician, which ensured multiple perspectives. Their varied backgrounds allowed for comprehensive insights into technical, practical, and organizational aspects, thereby collectively addressing all domains of the NASSS framework.

**Table 2.** Interview participants (N=13).

Background	Sex	Age
Project leader	Female (n=2) Male (n=3)	1 aged <30 y 2 aged 30‐39 y 1 aged 40‐49 y 1 aged ≥60 y
Physicians	Male (n=3)	2 aged 30‐39 y 1 aged ≥60 y
Nurse practitioner	Female (n=1)	1 aged 40‐49 y
Tele-nurse	Female (n=2)	2 aged 40‐49 y
Clinical informatician	Female (n=2)	1 aged <30 y 1 aged 40‐49 y

**Figure 1.**
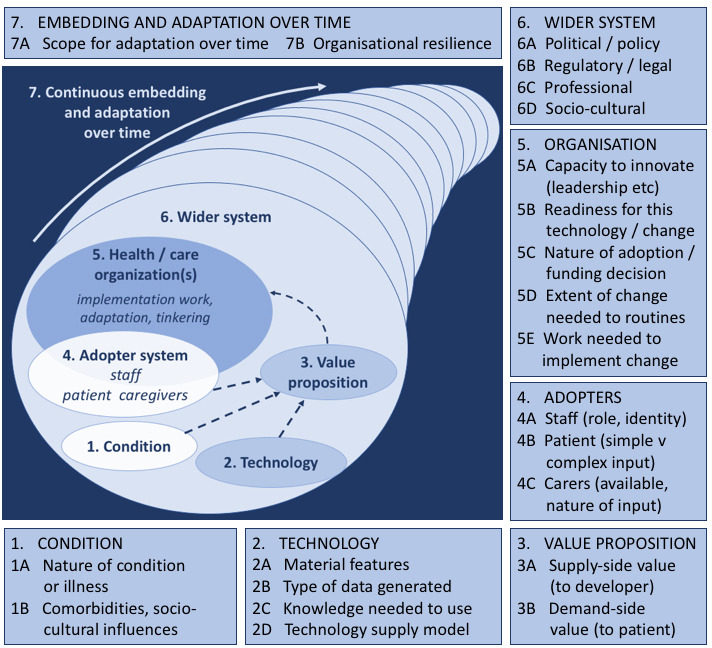
The non-adoption, abandonment, scale-up, spread, and sustainability (NASSS) framework [27].

Like previous studies that applied the NASSS framework to study telemonitoring (eg, 31,37-39), we based our topic list (see Multimedia Appendix 3) on this framework as well (see Figure 1) [27,40]. As the NASSS framework incorporates 7 interrelated domains that influence the implementation of innovative technologies, we considered it helpful to gain an understanding of relevant considerations of centralized versus (predominantly) distributed monitoring. The seven NASSS domains are as follows: (1) condition or illness, (2) technology, (3) value proposition, (4) adopter system, (5) organization, (6) wider context, and (7) embedding and adaptation over time [27]. Each domain can be defined by complexity (ie, dynamics, unpredictability, and interdependency) and their influence on other domains

The interviews were video recorded through Microsoft Teams and transcribed verbatim. Afterward, the transcripts were verified for accuracy by a single researcher (NAE). A 1-hour focus group was subsequently conducted with the 5 project leaders to reflect upon our findings and enrich our insights by discussing the findings. This focus group was audio-recorded and transcribed verbatim. The participants mainly recognized and affirmed the results, thereby supporting the validity of our findings.

### Data Analysis

The analysis was performed iteratively, ranging abductively between document analysis, transcripts (both interviews and focus group) and the literature [41,42]. A researcher (NAE) first open-coded the transcripts and documents via Atlas.ti 9 (Atlas.ti Scientific Software Development GmbH). To ensure reliability, a second researcher blindly thematically coded 3 interviews to cross-check coding. Afterward, the whole research team discussed both codes up to consensus, which resulted in a final code tree (see Multimedia Appendix 4), which guided a researcher (NAE) in the subsequent thematic coding [43]. Subsequently, selective coding categorized the codes and connected these to the themes of the NASSS framework domains. Next, each domain got a code, and the data was analyzed by these codes to identify similarities and differences across the cases, which led to an overview of complexities based on the criteria outlined in the NASSS framework. Finally, analysis and conclusions were discussed collectively (NAE, MJR, AMW, and MM). Overall, we applied a reflective thematic analysis as described by Braun and Clarke [43], “Reflexive TA [thematic analysis] approaches embrace researcher subjectivity as a resource for research (rejecting positivist notions of researcher bias (see [44]), view the practice of TA as inherently subjective, emphasize researcher reflexivity, and reject the notion that coding can ever be accurate—as it is an inherently interpretative practice, and meaning is not fixed within data.” Reflective thematic analysis was appropriate in this research, as we did not use the NASSS framework as a deterministic (ie, implementation steps) or even predictive model, rather as a sensitizing concept (see also abductive research methodology) providing us a reflexive lens in our analysis to deepen our interpretation [45].

## Results

This section presents the findings of this study, organized according to the 7 domains of the NASSS framework. For each domain, we compared the considerations made between centralized and distributed monitoring models.

### Domain 1: Selection Based on Patient Conditions and Telemonitoring Aims

The studied hospitals used various telemonitoring strategies: (1) centralized in the CMC, and (2) a predominantly distributed model in which most monitoring occurred locally, though some centralized elements were also present (see Table 1). The CMC strategy focused on identifying conditions suitable for central telemonitoring and aimed to incorporate all eligible patients into the CMC model. In contrast, in the distributed model, with the majority being managed through a distributed model, patients were selected on the basis of the telemonitoring objectives and disease complexity.

Our analysis revealed two objectives: (1) to enhance patient self-management, which is especially relevant in chronic diseases. Self-management was achieved through an online process involving telemonitoring that resembles e-coaching, in which advice is provided digitally, and hospital staff intervened only when the advice proved inadequate or when patients had questions. Since most alerts do not require an immediate response, distributed telemonitoring is feasible because it can be performed alongside other tasks. Several respondents mentioned that distributed telemonitoring was feasible for low-complexity cases only, because of time limitations; and (2) to monitor vital parameters. If patient measurements deviate from preset ranges, alerts are generated, prompting notifications to staff. Most alerts require immediate attention, as the patient may need acute care. Therefore, centralized telemonitoring aligns better with this aim.

### Domain 2: Technological Features and Number of Notifications

In the Netherlands, 2 main vendors offer telemonitoring software applications to hospitals (Luscii and Sananet). All studied hospitals used both applications along with several additional applications to answer patient questions, often as part of their patient portal or an independent phone app. Consequently, health care staff used several applications. The visual and architectural design of these applications differed across hospitals. Our data reveals no relationship between the choice of software applications and the organizational model for telemonitoring.

Instead, our findings indicate that the intended telemonitoring objectives (ie, Domain 1: self-management or vital parameter monitoring) and the work that needs to be done, especially the expected notification numbers, drive the selection of the organizational model. Vital parameter monitoring generates many alerts that demand a timely response. Alerts requiring urgent response impact heavily on the workload of the involved staff who must assess and filter alerts based on clinical relevance. Current software cannot distinguish between relevant and irrelevant alerts. The high volume of alerts cannot be managed alongside other tasks, necessitating either centralized monitoring or staff assigned to this function.

*The application captures most of the notifications or at least filters out the patients you don’t have to see because they are in good health. The CMC eliminates another 80%-90%. The nurses remove another 15% so that leaves the physician with zero to 5% patients to actually see.*

[Physician, hospital A]

In contrast, self-management provides less urgent alerts and, according to respondents, fewer alerts:

*Because the app encourages self-management, the number of alerts is very low […] We work in a team of three. I think about 1 FTE is assigned [to telemonitoring] for a population of more than 1000 patients.*

[Project leader, hospital B]

In sum, the technology setup is not tied to the organizational model. However, the aims of telemonitoring affect the expected volume of alerts and anticipated staff workload, which in turn influence the choice of organizational model.

### Domain 3: Influence of Efficiency and Knowledge on Value Propositions

Centralized and distributed telemonitoring offer distinct value propositions for both professionals and patients. The respondents indicated that in a centralized model, telemonitoring is assigned to dedicated tele-nurses in the CMC, allowing them to focus on telemonitoring tasks. Compared with a distributed model, their concentrated effort enables quicker response times compared to a distributed model (see Domain 2 subsection). Many respondents suggested that centralized telemonitoring operates more cost-effectively due to economy of scale. The benefits grow with more partnerships with other organizations or general practitioners (see Domain 5) and large numbers of patients are monitored. In contrast, respondents highlighted the cost-effectiveness of distributed telemonitoring models, as the telemonitoring tasks can be performed alongside other duties:

*[Nurses] in the outpatient clinic always have a spare moment between patients. They can answer a message. So, it is very energizing, and possible to do an ‘in-betweenie’. So, the question is, how much capacity do you win if you centralize this?*

[Project leader, hospital D]

In addition, our respondents said that delegating tasks to (tele-)nurses in both distributed and centralized monitoring alleviates the workload of nurse practitioners (NPs) and physicians, giving them more time for complex patient cases. However, task delegation sometimes requires specialist knowledge, especially with urgent vital parameter monitoring (see Domain 1 subsection). Specialist knowledge of many diseases is hard to transfer to CMC nurses and NPs, making it crucial for staff in centralized telemonitoring organizational models to have direct access to doctors. Respondents indicated that transferring specialized knowledge and maintaining close communication with doctors is easier to organize in distributed telemonitoring. Consequently, distributed telemonitoring models provide patients with direct access to health care professionals who possess specialist disease knowledge and, in some cases, specific knowledge about the patient. As one respondent said: “This specific knowledge adds value for patients.”

Thus, our data suggest that centralized telemonitoring is feasible for handling large patient and alert volumes (see Domains 1 and 2 subsections), which require nonspecialist knowledge, while distributed telemonitoring is preferable for alerts requiring specialist knowledge. Centralized telemonitoring is cost-efficient due to economies of scale when managing many patients or high volumes of alerts, whereas distributed telemonitoring is more efficient as it requires no additional personnel.

### Domain 4: Evolution of the Telemonitoring Adopter’s Role

In both organizational models, monitoring staff required specific competencies to incorporate the telemonitoring in their existing clinical workflows. Nurses took on a new role, expanded their responsibilities beyond those of traditional nursing. Consequently, the roles of NPs and physicians evolved, as now they supervise tele-nurses in their expanded responsibilities. In the cases we studied, the CMC tele-nurses were also responsible for developing telemonitoring clinical pathways for other medical conditions:

*I’m currently contacting physicians to determine which patient groups we can add to our monitoring program […] That’s my primary task besides monitoring. We also write the workflow protocols when we start monitoring a new condition.*

[Tele-nurse, hospital A]

Respondents emphasized that this prominent role in organizing telemonitoring contributed to the growing enthusiasm for the tele-nurse role, which led to fewer personnel shortages in the CMC compared to other departments.

In addition, tele-nurses are taking on tasks that were previously performed by physicians and nurse practitioners. This shift in responsibilities requires a strong foundation of trust—particularly in the clinical judgment, decision-making, and actions of tele-nurses. This is especially important when task distribution occurs across departmental boundaries, as is the case in centralized monitoring models. Central to establishing trust were collaboratively developed protocols, grounded in evidence-based medicine guidelines. This joint development process ensured continuity by actively involving the original care providers (eg, physicians and nurse practitioners) in shaping the clinical procedures. With this approach, confidence in the transfer of responsibilities was fostered and the transition of clinical tasks was supported. In addition, our data indicated that trust was established through several approaches: (1) making individualized care plans including patient-specific threshold for alerts, rather than relying on standard ranges for patients. This approach fostered the trust in clinical judgment of tele-nurses. (2) Developing new working routines, aligned with the task distribution. Clear communication about these new routines, particularly regarding responsibilities and alert management, helped build trust among professionals involved in the telemonitoring. (3) Project leaders fostered trust in centralized telemonitoring by facilitating disease-specific and training concerning the specific electronic medical record to tele-nurses, enabling a competent transition of care. Responsibilities were transferred to the CMC gradually, beginning with administrative tasks and progressively expanding to the full spectrum of telemonitoring activities, allowing trust to develop through demonstrated capability. Furthermore, respondents mentioned that staffing the CMC with nurses from diverse specializations ensured access to specialist knowledge, thereby increasing the confidence of colleagues in their ability to manage complex conditions and patients (Domains 2 and 3).

Thus, in both organizational models (tele-)nurses took on greater responsibility, under the supervision of NPs and physicians. Trust between professionals was crucial for this task redistribution, established through the development of evidence-based protocols, training, and clear guidelines, ensuring that (tele-)nurses could manage complex cases and alerts effectively.

### Domain 5: Telemonitoring Across Organizational Boundaries

Partnerships are a critical aspect of establishing CMC telemonitoring as efficiency depends on economy of scale. Collaborations can be either geographically bound (hospitals B and D) or based on previously established (national) network structures (hospital C). In our study, partnerships were primarily formed in centralized telemonitoring programs, likely due to the more structured coordination and larger scale of these programs compared to the more fragmented distributed models. Consequently, the objective function as a regional CMC appeared to be connected to their initial partner strategy. In contrast, hospital C is piloting a network-based collaboration also aimed at expansion. This network partnership involves complementary task delegation as the participants strive to delegate tasks to and from other organizations. Respondents also noted the potential for complementarity of regional and network collaboration, as the following quote shows:

*In the case of a deviating measure [of a patient’s vital signs], the CMC gets involved, prompting action. However, this action could potentially be delegated to a regional collaboration partner. The one does not rule out the other; they could work together very well.*

[Clinical informatician, hospital B]

Partnerships often allocate task delegation by (regional) partners to the CMC. However, respondents mentioned task delegation-related concerns about the freedom to comply or not with collaborative agreements and regional or network protocols. To address these concerns, hospitals B and D, having a majority of distributed telemonitoring cases, initially focused on regional initiatives, engaging all the partners (including GPs) in discussions on the challenges emerging from discrepancies between the protocols of the newly formed network and those of the established regional partnership.

To recap, partnerships are essential for CMCs to achieve efficiency through economy of scale. Collaborations can be regional or network-based, with CMCs benefiting from structured coordination, though task delegation between regional and network partners. However, this poses challenges related to protocol alignment and a need for making collaborative agreements.

### Domain 6: Economic Context Impedes Using Telemonitoring to Solve Societal Challenges

Our respondents aimed to provide efficient care in the face of staff shortages and the need to meet the increasing demand for care due to more patients with comorbidities and more elderly living at home. They feel telemonitoring can help address these challenges as it makes admitting patients or recurring outpatient clinic visits unnecessary. In the studied cases, a CMC employed nurses with physical limitations who were otherwise unable to provide direct patient care on the ward, demonstrating that telemonitoring allows these people to contribute to the workforce. By providing these new staff roles, telemonitoring, in both organizational models, supports workforce retention and efficiency.

In the Netherlands, both CMC and distributed telemonitoring are reimbursed under the same scheme. However, the current financing model within the diagnosis treatment combination (similarities with DRG’s systems) system is predicated on the substitution of physical care with remote care within the existing tariff structure. In practice, reimbursement rates for in-person care remain higher than those for remote services. Furthermore, hospitals are required to make substantial upfront investments in digital infrastructure and personnel to support telemonitoring and remote care services. As a result, costs are incurred in advance of potential long-term benefits, and providers risk losing revenue from reduced volumes of billable physical consultations and hospital admissions. Given that hospital funding in the Netherlands is still partially based on production-related agreements—largely tied to physical service delivery—this shift toward more efficient, home-based care may inadvertently lead to a decline in institutional revenue, thereby creating a financial disincentive for scaling up telemonitoring:

*We have lots of telemonitoring and that costs us millions of euros [because we don’t get fully reimbursed]. So, you’re always thinking, what am I doing? I’m organizing my own downfall.*

[Project leader, hospital A]

Since there is no difference in reimbursement for centralized and distributed telemonitoring, these financial challenges are the same for all hospitals in this study.

### Domain 7: Embedding and Adapting Telemonitoring Over Time

The CMCs in all hospitals are currently expanding, and many respondents anticipate an even broader role for telemonitoring in the future. Expected developments include more partnerships in CMC, extending the number of patient conditions monitored, and increased patient volumes.

*We’re expanding to monitor more care pathways, and we’re also aiming to increase the number of patients on each pathway.*

[Project leader 1, hospital C]

Some respondents suggest that advancements in information and communication technologies and artificial intelligence (AI) could facilitate this role expansion, especially in automating tasks such as distinguishing relevant from irrelevant notifications. This is particularly applicable to acute-care patients who require intensive monitoring. However, if automation leads to reduced numbers of notifications, it could also reduce the need for centralization.

### NASSS Framework

**Figure 2.**
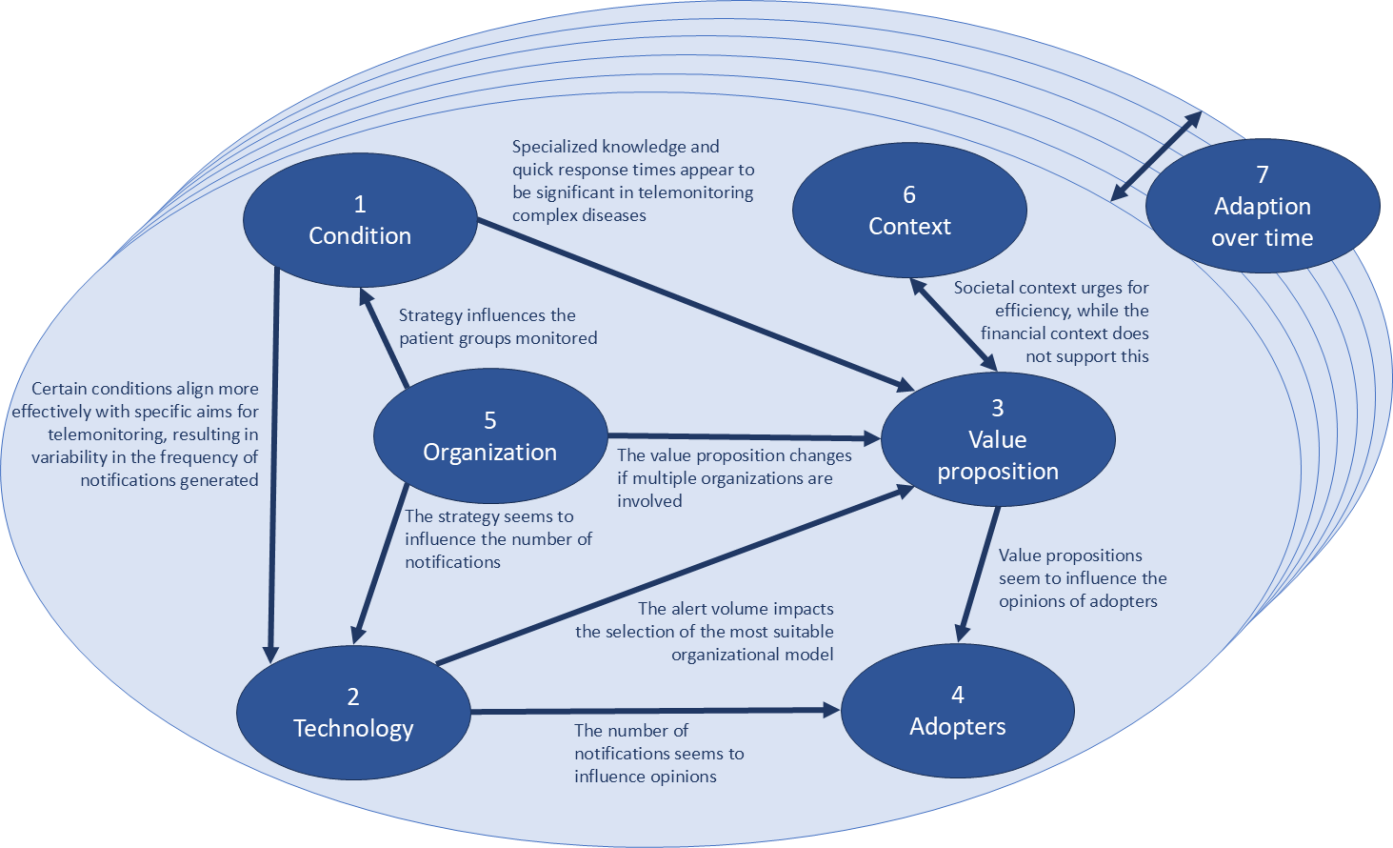
Interactions between the domains of the non-adoption, abandonment, scale-up, spread, and sustainability (NASSS) framework [27].

Using the NASSS framework enabled us to examine the domains while considering multiple decision-making factors. Figure 2 illustrates the interdependencies found among the various domains. For instance, value proposition (Domain 3) is primarily based on the perception of cost-effectiveness and added value to the patient. It is also affected by the selected patient group (Domain 1) and organizational strategy (Domain 5). The technology applied (Domain 2) affects how notifications are managed and handled, impacting the value proposition and opinion of adopters. When changes made in an area can affect other areas, interconnectedness underscores the complexity of decision-making in organizing telemonitoring.

## Discussion

### Principal Findings

This multiple case study shows that the decision to implement telemonitoring, whether through centralized or distributed organizational models, involves a complex array of considerations that must be carefully weighed. A definitive argument in favor of either model cannot be made, as each option presents distinct advantages and challenges across different domains. However, our findings highlight that strategic decisions made early in the implementation process—particularly those related to the telemonitoring of objectives on the basis of patient groups—are crucial in determining the appropriate organizational model. These decisions, in turn, shape possibilities related to personnel (eg, hiring tele-nurses or educating outpatient clinic or ward staff, and the role physicians play), partnerships, and the overall cost-effectiveness of telemonitoring.

### Comparison With Previous Work

To date, many telemonitoring studies have focused on the design and development of the technology or the implementation of a specific telemonitoring care pathway [10-12,46]. To our knowledge, this is the first study to systematically compare considerations related to centralized and distributed telemonitoring models.

#### Model Follows Strategy

First, we would like to address the significant role of the selected patient condition, encompassing both the type of patient group enrolled in telemonitoring and the complexity of their health care needs. The patient condition drives the subsequent considerations that together determine the appropriate organizational telemonitoring model. For example, programs targeting chronic conditions aiming for self-management, typically associated with lower alert frequency and lower clinical complexity, can be organized through distributed care models. Conversely, telemonitoring programs focused on vital parameter tracking, such as those in early discharge scenarios [10,23,24,47], having high alert volumes and a need for rapid response, make centralized models more appropriate in those cases (domains 1 and 2). This observation is consistent with the current literature, which suggests that before implementation, telemonitoring systems are evaluated for their anticipated impact on patient safety, staff workload, and clinical usefulness [24,48], all of which depend on the specific patient group intended for remote care. This initial strategic decision sets the foundation for the primary objective of telemonitoring, whether that be to enhance self-management [48,49] or to monitor vital parameters [50]. Consequently, the selected patient condition and objectives influence the aims, and vice versa. Furthermore, the chosen aims directly affect the volume of alerts generated. For instance, a focus on vital parameter monitoring typically results in a greater frequency of alerts [51] compared to a self-management approach. Our findings indicate that the volume of alerts also affects how and for whom notifications are managed, ultimately shaping workload distribution [52] and task division [53,54] among health care professionals. Clearly, the interplay between patient condition, strategic objectives, and alert management is critical in determining the appropriate organizational model for telemonitoring. The choice of objectives impacts personnel, capacity, resources, and partnerships, outcomes that affect health care delivery in the region. It is crucial not to overlook the need for planning at this level, and decision makers must be educated to consider all the dimensions and understand how their choices will ripple through the system when determining the best telemonitoring model for a patient. Looking ahead, the evolving role of AI will likely have significant implications for the organization of telemonitoring (Domain 7). AI has the potential to support clinical decision-making by filtering alerts, identifying patterns, and prioritizing cases [55]. In this way, AI may reduce the need for centralized monitoring by enabling distributed care teams to manage alerts more efficiently alongside their routine tasks. Conversely, the increasing amount of data and complexity requires centralized systems, where specialized staff works with AI to manage the increasing data streams. While the direction of this impact remains uncertain, these technological developments will influence the suitability of different organizational models.

#### Personnel

In our case studies, the respondents’ perceptions of the effectiveness of telemonitoring and its added value for patients played a significant role in choosing a model. Even in the absence of clear evidence supporting the superiority of one model over another, in terms of cost-effectiveness or patient benefit, respondents based their preference on subjective evaluations [4,56,57]. This is notable, particularly given that the attitudes of health care professionals (eg, nurses and physicians) are a significant factor in the acceptance of telemonitoring in practice [24], and these professionals are regarded as the “most important gatekeepers for telehealth services” [58]. Thus, the attitudes and perceptions of health care professionals influence the implementation of telemonitoring, impacting not only local adoption but also broader upscaling [54]. Health care professionals also play an important role in deciding on the choice of telemonitoring aims in the process of selecting a suitable model. This makes it essential that their decisions are not based solely on subjective judgments. Rather, they should be well-informed about the rationale behind selecting specific telemonitoring models.

In addition, implementing telemonitoring, whether centralized or distributed, is driven by the objective of increasing efficiency and addressing staff shortages [2,4]. As patients increasingly adopt self-management techniques [59,60], and health care services evolve, the roles and tasks of health care professionals are undergoing significant transformation. The literature supports this shift, indicating that telemonitoring influences task division in health care organizations [61], resulting in the creation of new roles and responsibilities [62] while potentially disrupting established professional roles [63]. Given this knowledge, organizations must align their personnel preparation and organizational models accordingly. It is essential to equip staff for new responsibilities through targeted training programs that address the specific skills needed for telemonitoring [64,65]. In addition, our findings show that the redistribution of tasks, particularly when tele-nurses assume responsibilities previously handled by physicians, was eased by collaborative efforts in developing protocols and workflows. Finally, the division of created tasks should be compatible with the organizational model and available resources. Therefore, when selecting a telemonitoring model, organizations should consider their capacity, resources, and personnel.

#### Partnerships, Collaborations, and Networks

Our findings place key emphasis on the importance of partnerships and collaborations in organizing telemonitoring, particularly in the centralized monitoring context, although both models may involve multiple organizations [13,26]. The participants acknowledged both the potential benefits of working with various partners and the challenges associated with delegating tasks among health care providers. However, the discussion focused on the division of tasks between providers and the clinical pathway design, while the complexities of interorganizational collaboration remained largely unaddressed. This oversight parallels a notable gap in the literature on organizing telemonitoring collaboratively. Defining responsibilities, establishing safety protocols, streamlining workflows, sharing knowledge, and educating personnel are all important when multiple organizations participate in telemonitoring [24]. While some studies touch upon interorganizational collaboration among health care providers [24], there is still insufficient exploration of the necessary governance and management frameworks required for telemonitoring collaboration in practice [66,67]. This is concerning from a quality perspective, given that effective collaboration between care providers is essential for ensuring continuity of care [46]. Scholars suggest that successfully scaling telemonitoring on a regional or national level hinges on resources and reimbursement structures [54]. Both elements should be discussed when collaboratively organizing telemonitoring. Given that network theory offers valuable insights into managing networks with common goals, in this case regional telemonitoring implementation, we want to emphasize that scholars should not reinvent the wheel when there is a compelling opportunity to use this knowledge to navigate organizational boundaries effectively [68].

### Strengths and Limitations

From a practical perspective, this research contributes to a deeper understanding of the decision-making processes involved in telemonitoring services. The insights derived from this study offer valuable guidance for health care organizations seeking to implement or scale telemonitoring programs, or to design effective telemonitoring pathways with regional partners. From a theoretical standpoint, this study extends the application of the NASSS framework by examining organizational-level decision making across all NASSS domains. In doing so, we illustrate how the various domains interact and influence one another in the context of organizing telemonitoring. We acknowledge, however, three primary limitations. (1) While the NASSS framework provides a comprehensive approach to integrating diverse perspectives on implementing eHealth technology, these categories may constrain data interpretation. Specifically, the framework tends to overlook the relational dynamics, for instance, between caregivers and patients, despite the literature that supports the importance of including human interactions in implementation [48]. Future scholars could benefit from using NASSS-CAT tools [40]. This combination allows for a theory-informed framework, supplemented by a pragmatic planning tool. (2) The limited exploration of ethical-legal considerations and data privacy in the context of telemonitoring may be attributed to the specific respondent group chosen for inclusion. Issues related to data sharing, patient consent, and privacy must be addressed to ensure the ethical deployment of telemonitoring technologies [24]. These legal and ethical topics should be prioritized in future research to provide a more comprehensive understanding of the implications of telemonitoring on patient data and privacy rights. Another limitation lies in (3) the aim to contrast a centralized model with a fully distributed one. However, in practice, such clear-cut models are hard to find. As a result, we included 2 cases using predominantly distributed models, where respondents also had experience with centralized approaches or used centralized models for specific clinical pathways. Although this hybrid reality (literature) reflects the complexity of studying real-world implementation, it blurs the distinction between the two models.

### Conclusions

This study provides insights into the complexities of decision-making involved in organizing telemonitoring and offers guidance to all stakeholders, including health care professionals, on understanding the considerations and consequences of the choices. It identifies specific rationales for selecting either a centralized or distributed telemonitoring model, depending on patient needs, such as those requiring vital parameter monitoring or self-management. The volume of notifications generated also is a critical factor in determining the most suitable model. In addition, perceptions of efficiency and the perceived added value to patients significantly influence decision-making. The study underscores the importance of considering technological, organizational, and societal factors when choosing between centralized and distributed models. Notably, decisions made in early stages, primarily by physicians, can have far-reaching implications for personnel, resources, and partnerships at a regional level. When starting out with telemonitoring, those in charge should recognize the interconnectedness of factors and carefully consider all seven NASSS domains.

## Supplementary material

10.2196/69349 Multimedia Appendix 1 Visualization of a centralized organizational model (example).

10.2196/69349 Multimedia Appendix 2 Visualization of a distributed organizational model (example).

10.2196/69349 Multimedia Appendix 3 Topic list.

10.2196/69349 Multimedia Appendix 4 Codetree (in Dutch).

10.2196/69349 Checklist 1 COREQ checklist.

## Abbreviations

AI: artificial intelligence
CMC: centralized monitoring center
COPD: chronic obstructive pulmonary disease
COREQ: Consolidated Criteria for Reporting Qualitative Research
NASSS: non-adoption, abandonment, scale-up, spread, and sustainability
NP: nurse practitioner
