# Supplementary figures and images for: Organizing Telemonitoring—Decision-Making Between Centralized and Distributed Models in the Netherlands, Using the Non-Adoption, Abandonment, Scale-Up, Spread, and Sustainability (NASSS) Framework: Case Study
Source: JMIR Med Inform. 2025 Oct 8;13:e69349. doi: 10.2196/69349 (PMC12507340; doi:10.2196/69349)

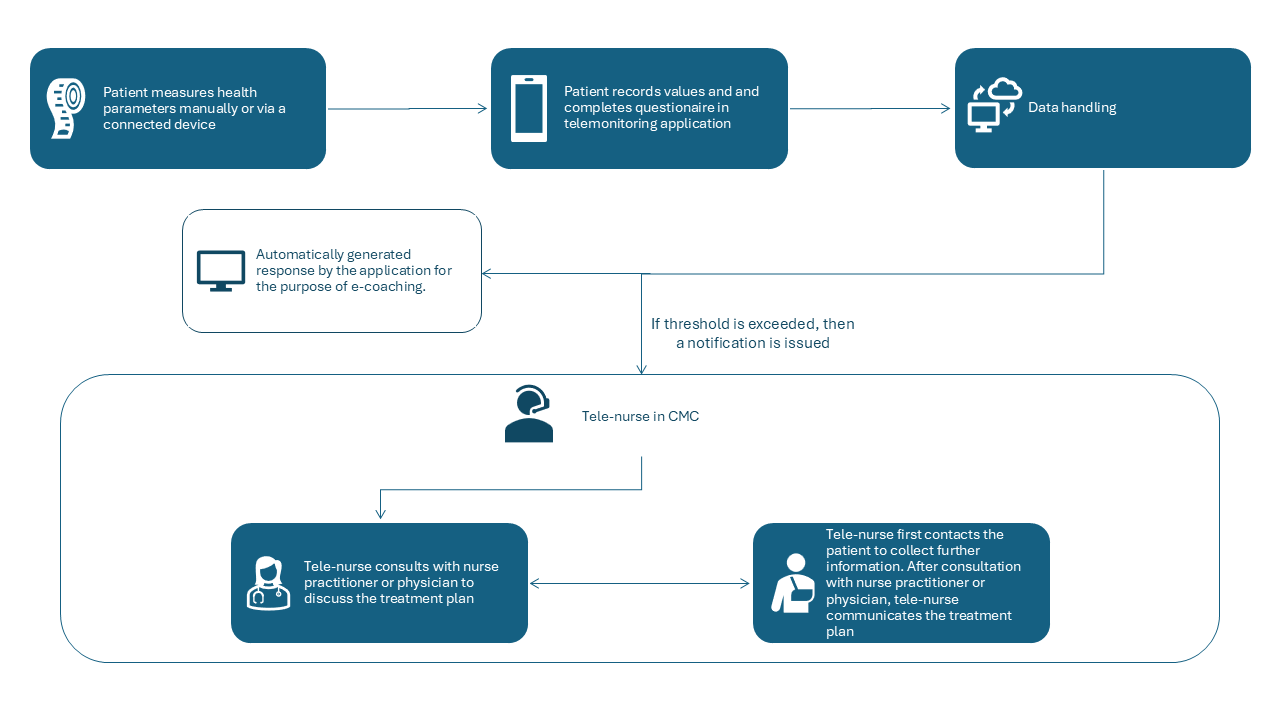

Supplement: Multimedia Appendix 1 [file medinform-v13-e69349-s001.PNG]

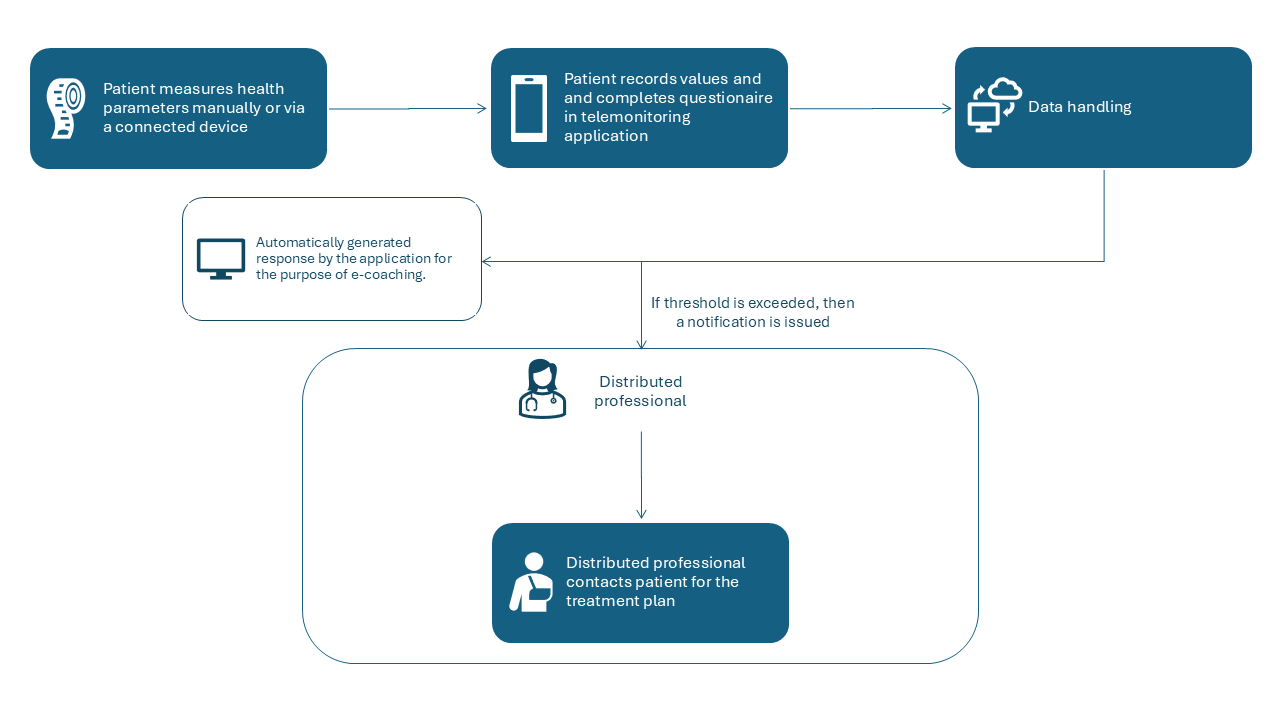

Supplement: Multimedia Appendix 2 [file medinform-v13-e69349-s002.PNG]

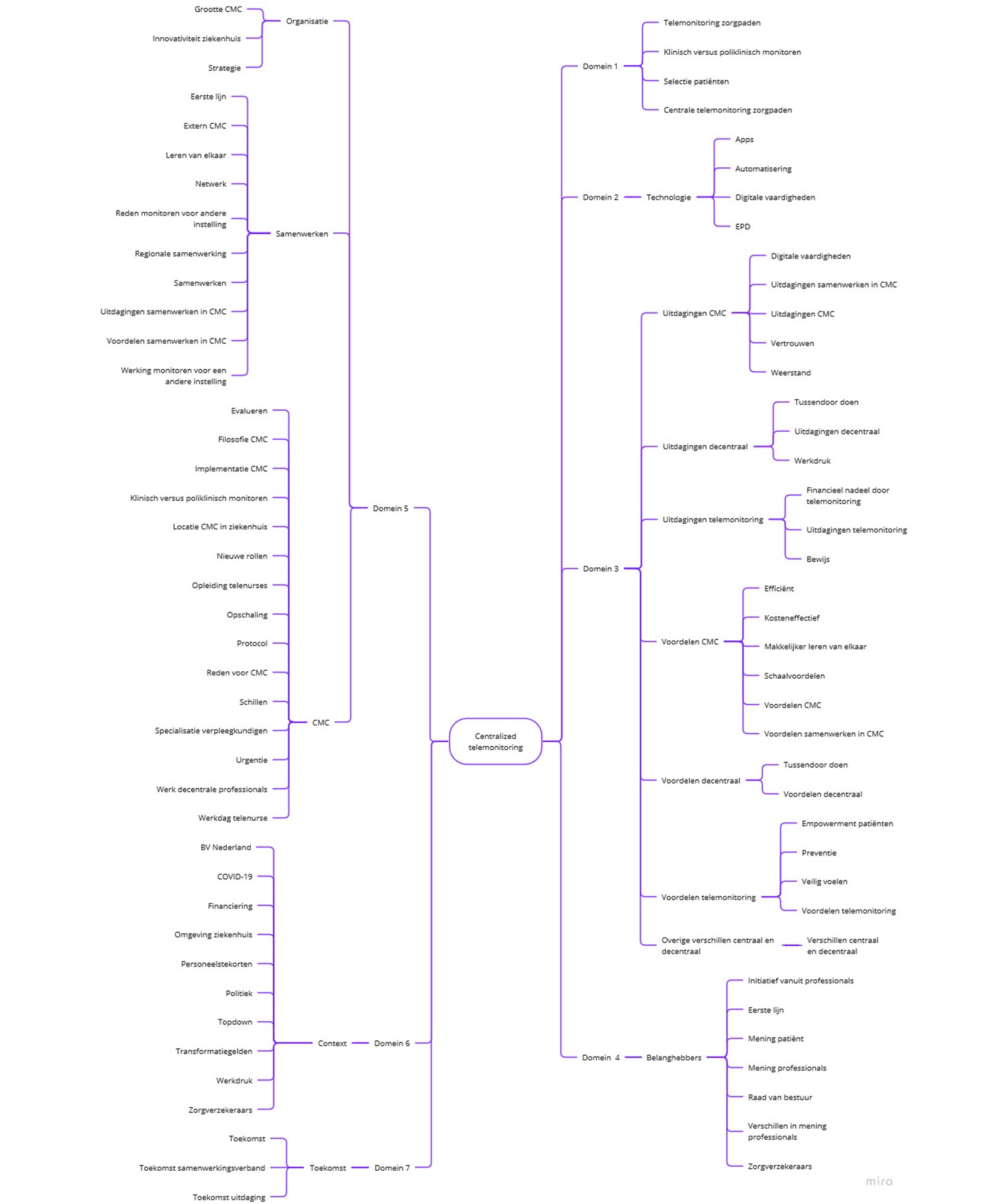

Supplement: Multimedia Appendix 4 [file medinform-v13-e69349-s004.png]
