# Supplementary material for: Organizing Telemonitoring—Decision-Making Between Centralized and Distributed Models in the Netherlands, Using the Non-Adoption, Abandonment, Scale-Up, Spread, and Sustainability (NASSS) Framework: Case Study
Source: JMIR Med Inform. 2025 Oct 8;13:e69349. doi: 10.2196/69349 (PMC12507340; doi:10.2196/69349)
Supplement: Multimedia Appendix 3 [file medinform-v13-e69349-s003.docx]

# Appendix 4: Topic List

## Project Leaders

### Introduction

- Who are you?
- What is your role in this organization?
- What is your background?

### General Information

- Why did you choose a centralized monitoring center?
- Are all telemonitoring care pathways managed centrally? If not, why are certain pathways managed centrally while others are not?
- Which care pathways are managed centrally? Why?
- How far along are you with the implementation of centralized telemonitoring?
- How many patients are currently participating in your centralized telemonitoring program?
- How does the telemonitoring technology integrate with other electronic systems in the hospital, such as the Electronic Patient Record (EPR)? If it doesn’t, why not?

### Collaboration Structure

- Why did you choose to monitor for other hospitals/general practitioners (GPs) as well?
- How is the collaboration arranged with the other hospitals/GPs?
- What is the relationship with the other hospitals/GPs in the collaboration?
- Who is ultimately responsible for the care of patients from other hospitals/GPs, for example, if an error occurs?

### Implementation Process

- Were you already using distributed telemonitoring before implementing centralized telemonitoring?
- How did you start implementing centralized telemonitoring?
- Is implementing centralized telemonitoring any different than implementing telemonitoring in a single department? In what way(s)?

### The Value of Telemonitoring

- How effective is centralized telemonitoring?
- Are there any differences in effectiveness compared to distributed telemonitoring? What are they, and why?
- What impact does centralized telemonitoring have on patient safety compared to distributed monitoring?
- What are the benefits of centralized telemonitoring compared to distributed telemonitoring?
- What are the challenges of centralized monitoring compared to distributed?
- What are the advantages of distributed monitoring?
- What are the challenges of distributed monitoring?

### Changes Resulting from Centralized Telemonitoring

- What impact does the central monitoring center have on staffing distributed personnel?
- What qualities [competences] do personnel require to work in the monitoring center?
- To what extent does implementing this technology change the work of distributed doctors and nurses?
- What has changed in the role of doctors and nurses, in your opinion?
- What feedback have you received from nurses and doctors on centralized telemonitoring? What do you think of this feedback?
- What does centralized telemonitoring change in existing care pathways?
- Who is ultimately responsible for the care of your own hospital's patients, for example, if an error occurs?
- What feedback have you received from patients on centralized telemonitoring?
- How is centralized telemonitoring different for patients compared to telemonitoring by a single department or institution?

### Finance

- How is the centralized monitoring center funded?
- How is the funding different than telemonitoring in a single hospital or department?
- How cost-effective is centralized telemonitoring? Is this different than distributed telemonitoring?

### Context

- To what extent does centralized telemonitoring align with your hospital’s strategy?
- How innovative is your hospital compared to other hospitals?
- What influence does politics have on the implementation of centralized telemonitoring?
- What influence do laws and regulations have on the implementation?
- What influence does the network you are part of have on the implementation?
- To what extent does your operating environment (location, etc.) influence the implementation?
- How does the context influence the implementation of centralized versus regular telemonitoring?

### Future

- How do you see centralized telemonitoring developing over time?
- What challenges do you foresee in the future?

## Physicians and Nurse Practitioners

### Introduction

- Who are you?
- What is your role in this organization?
- What is your background?

### General Information

- How is centralized telemonitoring used? Which care pathways involve centralized telemonitoring? How are those care pathways selected?
- Why are certain care pathways managed centrally while others are not?

### Implementation Process

- How did you find the process of implementing centralized telemonitoring? What went well, and what didn’t?
- What changes were necessary to implement centralized telemonitoring?
- Why did you choose to organize telemonitoring centrally? And why is telemonitoring in other departments distributed?

### The Value of Telemonitoring

- How effective is centralized telemonitoring?
- Is the effectiveness of centralized telemonitoring any different compared to distributed telemonitoring? What are the differences, and why?
- What impact does centralized telemonitoring have on patient safety compared to distributed monitoring?
- What are the benefits of centralized telemonitoring compared to distributed telemonitoring?
- What are the challenges of centralized monitoring compared to distributed?
- What are the advantages of distributed monitoring?
- What are the challenges of distributed monitoring?

### Change

- What do you think of centralized telemonitoring? How does it differ from distributed monitoring by either the patient’s own healthcare provider or an external office?
- What impact does the central monitoring center have on your department?
- How has implementing this technology changed your work? How would this differ from decentralized telemonitoring?
- How has centralized telemonitoring changed your role?
- How is the collaboration between the original care providers and the monitoring center?
- What changes does centralized telemonitoring bring to the existing care pathways?
- Does the telemonitoring technology integrate with other electronic systems in the hospital, such as the EHR (Electronic Health Record)? If not, how does this affect your work?
- Who is ultimately responsible for the care of the patients in your hospital, for example, if an error occurs?
- Is centralized telemonitoring used for all patients? If not, what criteria are used for selection? Is this different than distributed telemonitoring?
- What feedback have you received from patients on centralized telemonitoring?
- How does centralized telemonitoring differ for patients compared to distributed monitoring?

### Future

- How do you see centralized telemonitoring developing over time?
- What challenges do you foresee in the future?

## Tele-nurses

### Introduction

- Who are you?
- What is your role in this organization?
- What is your background?

### General Information

- What is your role in the care pathway?
- What is your work like? How is it different than working in a department?
- Do you provide care for various conditions? If so, how do you feel about this?
- Does the telemonitoring technology integrate with other electronic systems in the hospital, such as the EHR (Electronic Health Record)? If not, how does this affect your work?
- Is centralized telemonitoring used for all patients? If not, what criteria are used for selection?
- Why are certain care pathways managed centrally while others are not?
- Do you monitor patients in need of clinical care or patients in need of outpatient care?
- How is centralized telemonitoring used? Which care pathways involve centralized telemonitoring? How are those care pathways selected?
- How did you find the process of implementing centralized telemonitoring? What went well, and what didn’t?
- What changes were necessary to implement centralized telemonitoring?

### The Value of Telemonitoring

- How effective is centralized telemonitoring?
- Are there any differences in effectiveness compared to distributed telemonitoring? What are they, and why?
- What impact does centralized telemonitoring have on patient safety compared to distributed monitoring?
- What are the benefits of centralized telemonitoring compared to distributed telemonitoring?
- What are the challenges of centralized monitoring compared to distributed?
- What are the advantages of distributed monitoring?
- What are the challenges of distributed monitoring?

### Change

- What do you think of centralized telemonitoring?
- How do you find working with centralized telemonitoring?
- To what extent has implementing this technology changed your work compared to your previous job?
- What do other, distributed healthcare staff think of centralized telemonitoring?
- How is the collaboration between the original care providers and the monitoring center?
- What changes does centralized telemonitoring bring to the existing care pathways?
- Who is ultimately responsible for the care of the patients in your hospital, for example, if an error occurs?
- What feedback have you received from patients on centralized telemonitoring?
- How does centralized telemonitoring differ for patients compared to distributed monitoring?

### Future

- How do you see centralized telemonitoring developing over time?
- What challenges do you foresee in the future?

## Distributed Physicians and Nurse Practitioners

### Introduction

- Who are you?
- What is your role in this organization?
- What is your background?

### General Information on Telemonitoring

- How is centralized telemonitoring used? Which care pathways involve centralized telemonitoring? How are those care pathways selected?
- Why are certain care pathways managed centrally while others are not?

### Implementation Process

- How did you find the process of implementing telemonitoring? What went well, and what didn’t?
- What changes were necessary to implement telemonitoring?
- Why did you choose to implement distributed telemonitoring? And why is telemonitoring in other departments still centralized?

### The Value of Telemonitoring

- How effective is distributed telemonitoring?
- Are there any differences in effectiveness compared to centralized telemonitoring? What are they, and why?
- What impact does distributed telemonitoring have on patient safety compared to centralized monitoring?
- What are the advantages of distributed monitoring?
- What are the challenges of distributed monitoring?
- What are the benefits of centralized telemonitoring compared to distributed telemonitoring?
- What are the challenges of centralized monitoring compared to distributed?

### Change

- What do you think of distributed telemonitoring? How does it differ from centralized monitoring? Or monitoring by an external office?
- What impact does the use of telemonitoring have on your department?
- How has implementing this technology changed your work? How might this differ from centralized telemonitoring?
- How has distributed telemonitoring changed your role?
- Is the telemonitoring technology integrated with other electronic systems in the hospital, such as the EHR (Electronic Health Record)? If not, how does this affect your work?
- Who is ultimately responsible for the care of the patients in your hospital, for example, if an error occurs?
- Is telemonitoring used for all patients? If not, what criteria are used for selection? Would this be different than centralized telemonitoring?
- What feedback have you received from patients on telemonitoring?
- How is distributed telemonitoring different for patients compared to centralized telemonitoring?

### Future

- How do you see centralized telemonitoring developing over time?
- What challenges do you foresee in the future?

## Clinical Informaticians

### Introduction

- Who are you?
- What is your role in this organization?
- What is your background?

### General Information

- Why did you choose a centralized monitoring center?
- Are all telemonitoring care pathways managed centrally? If not, why are certain pathways centrally managed while others are not?
- Which care pathways have you centralized? Why?
- How does the telemonitoring technology integrate with other electronic systems in the hospital, such as the EHR (Electronic Health Record)? If it doesn’t integrate, why not?
- Which apps do you use? What functions do these different apps provide?

### Collaboration Structure

- What impact do the apps have on collaboration between the centralized monitoring center and distributed professionals?
- Can you access the EHR of the other hospital(s) or general practitioners? If not, why not? What are the consequences of this?

### Implementation Process

- Were you already using distributed telemonitoring before implementing centralized telemonitoring?
- How did you start implementing centralized telemonitoring? What were the setbacks? What went well?
- What changes were necessary to implement centralized telemonitoring?
- What technical challenges did you encounter?
- How does implementing centralized telemonitoring differ from implementing distributed telemonitoring?
- How does the process of implementing a new care pathway work?
- What impact does centralization have on technology?
- What technical challenges and complications arise during the implementation of telemonitoring?

### The Value of Telemonitoring

- How effective is centralized telemonitoring?
- Are there any differences in effectiveness compared to distributed telemonitoring? What are they, and why?
- What impact does centralized telemonitoring have on patient safety compared to distributed monitoring?
- What are the benefits of centralized telemonitoring compared to distributed telemonitoring?
- What are the challenges of centralized monitoring compared to distributed?
- What are the advantages of distributed monitoring?
- What are the challenges of distributed monitoring?

### Context

- What influence does politics have on the implementation of centralized telemonitoring?
- What influence do laws and regulations have on the implementation?
- To what extent does the operating environment (location, etc.) influence the implementation?
- How does the context influence the implementation of centralized versus regular telemonitoring?

### Future

- How do you see centralized telemonitoring developing over time?
- What challenges do you foresee in the future?

## Focus Group

- To what extent do you recognize yourself in the results?
- Do you recognize yourself in the different implementations that hospitals have for their monitoring centers?
- To what extent do you see this reflected in practice?
- How do you see the tension between regional and (internal) network collaboration?
